# Einsatzmöglichkeiten digitaler Tools in der postoperativen Schmerztherapie

**DOI:** 10.1007/s00482-023-00732-7

**Published:** 2023-07-10

**Authors:** Jana L. Aulenkamp, Lina Mosch, Christine H. Meyer-Frießem, Nathalie M. Malewicz-Oeck

**Affiliations:** 1 Klinik für Anästhesiologie und Intensivmedizin, Universitätsklinikum Essen, Universität Duisburg-Essen, Hufelandstr. 55, 45122 Essen, Deutschland; 2 Klinik für Anästhesiologie mit Schwerpunkt operative Intensivmedizin, Charité – Universitätsmedizin Berlin, corporate member of Freie Universität Berlin and Humboldt-Universität zu Berlin, Berlin, Deutschland; 3 Institut für Medizinische Informatik, Charité – Universitätsmedizin Berlin, corporate member of Freie Universität Berlin and Humboldt-Universität zu Berlin, Berlin, Deutschland; 4 Klinik für Anästhesiologie, Intensiv- und Schmerzmedizin, Universitätsklinikum Bergmannsheil Bochum gGmbH, Bochum, Deutschland; 5 Klinik für Anästhesiologie, Intensiv- und Schmerzmedizin, St. Marien Hospital, Lünen, Deutschland

**Keywords:** Chronischer Schmerz, Akuter Schmerz, eHealth, Schmerzmanagement, Digitalisierung, Chronic pain, Acute pain, ehealth, Pain management, Digitalization

## Abstract

**Hintergrund:**

In letzter Zeit finden zunehmend digitale Tools wie Smartphone-basierte Applikationen und der Einsatz künstlicher Intelligenz Einzug in die Schmerzmedizin. Dies könnte im postoperativen Schmerzmanagement neue Therapieansätze ermöglichen. Der vorliegende Beitrag gibt einen Überblick über verschiedene digitale Tools und deren Einsatzmöglichkeiten in der postoperativen Schmerztherapie.

**Material und Methoden:**

Es wurde eine orientierende Literaturrecherche in den Datenbanken MEDLINE und Web of Science durchgeführt und eine gezielte Auswahl von Publikationen getroffen, um eine strukturierte Darstellung verschiedener aktueller Einsatzmöglichkeiten vorzunehmen und auf Basis neuester Erkenntnisse zu diskutieren.

**Ergebnisse:**

Heute gehören zu den Einsatzmöglichkeiten digitaler Tools – wenn auch meist nur mit Modellcharakter – die Schmerzdokumentation und -erfassung, das Selbstmanagement sowie die Edukation der Patient:innen, die Schmerzprädiktion, Entscheidungsunterstützung für das Fachpersonal sowie die supportive Schmerztherapie, beispielsweise in Form von virtueller Realität und Videos. Dies bietet Vorteile wie individualisierte Behandlungskonzepte, das Adressieren bestimmter Patient:innengruppen, Reduktion von Schmerzen und Analgetika sowie das Potenzial der Frühwarnung oder -erkennung von postoperativen Schmerzen. Im vorliegenden Beitrag werden ebenso die Herausforderungen der technischen Umsetzung und angemessenen Schulung der Nutzer:innen thematisiert.

**Schlussfolgerung:**

Der Einsatz digitaler Tools, wenngleich bisher eher punktuell und modellhaft im klinischen Alltag integriert, verspricht zukünftig eine innovative, personalisierte postoperative Schmerztherapie. Künftige Studien und Projekte sollten dazu beitragen, die vielversprechenden Forschungsansätze in den klinischen Alltag zu integrieren.

**Zusatzmaterial online:**

Die Online-Version dieses Beitrags (10.1007/s00482-023-00732-7) enthält eine Tabelle mit den eingeschlossenen Studien.

## Abstract (en)

**Background:**

Recently, digital tools, such as smartphone-based applications and the use of artificial intelligence have increasingly found their way into pain medicine. This could enable new treatment approaches in postoperative pain management. Therefore, this article provides an overview of various digital tools and their potential application options in postoperative pain management.

**Material and methods:**

An orienting literature search was carried out in the MEDLINE and Web of Science databases, and a targeted selection of essential key publications was made in order to provide a structured presentation of different current possible applications and a discussion based on the most recent knowledge.

**Results:**

Today, possible applications of digital tools, even if they mostly have only a model character, include pain documentation and assessment, patient self-management and education, pain prediction, decision support for medical staff, and supportive pain therapy, for example in the form of virtual reality and videos. These tools offer advantages such as individualized treatment concepts, addressing specific patient groups, reduction of pain and analgesics, and the potential for early warning or detection of postoperative pain. Furthermore, the challenges of the technical implementation and appropriate user training are highlighted.

**Conclusion:**

The use of digital tools, although so far integrated in clinical routine in a relatively selective and exemplary manner, promises to be an innovative approach for personalized postoperative pain therapy in the future. Future studies and projects should help to integrate the promising research approaches into everyday clinical practice.

Digitale Anwendungen finden zunehmend Einzug in die Medizin. Unter den bisher eingeführten digitalen Gesundheitsanwendungen, den Therapie-Apps, deren Einsatz von Krankenkassen erstattet wird, sind bereits einige Anwendungen auf Schmerzerkrankungen ausgerichtet. Seit der Coronavirus-disease-2019(COVID-19)-Pandemie werden ebenfalls verstärkt digitale Tools eingesetzt. Doch wie sehen die aktuellen digitalen Einsatzmöglichkeiten in der postoperativen Schmerztherapie aus? Gibt es neue Ansätze, um den nach wie vor gravierenden Herausforderungen von Diagnostik, Therapie und Prävention der akuten und chronischen postoperativen Schmerzen zu begegnen?

## Herausforderungen postoperativer Schmerzen

Akute postoperative Schmerzen (APS) gehen teils mit hohen Schmerzintensitäten einher [11]. Folgend chronifizieren diese je nach Eingriff häufig [25]. Beispielsweise entwickeln etwa 45 % der Patient:innen nach chirurgisch versorgten Frakturen chronische postoperative Schmerzen (CPS; [1]). Die Ätiologie postoperativer Schmerzen ist vielfältig, und zu den bekannten Risikofaktoren zählen beispielsweise ein niedriges Lebensalter oder vorbestehende chronische Schmerzen [11, 25]. Da die Lebensqualität der Betroffenen stark reduziert ist, werden die Mechanismen der Entstehung und Aufrechterhaltung postoperativer Schmerzen sowie wirksame Behandlungskonzepte seit Jahren intensiv untersucht.

Digitale Tools sind bislang kaum im Klinikalltag der postoperativen Schmerztherapie integriert. Noch vor fünf Jahren wurden in Kanada lediglich zehn Apps für das postoperative Schmerzmanagement identifiziert, von denen 80 % der reinen Edukation dienten und keine für das Selbstmanagement oder die Behandlung mit evidenzbasierten Vorschlägen konzipiert war [16]. Auf dem Weg zur Optimierung der postoperativen Schmerztherapie arbeiten interdisziplinäre Forschungsteams zunehmend an der Entwicklung neuer digitaler Konzepte für diesen Bereich.

## Material und Methoden

**Figure 1:**
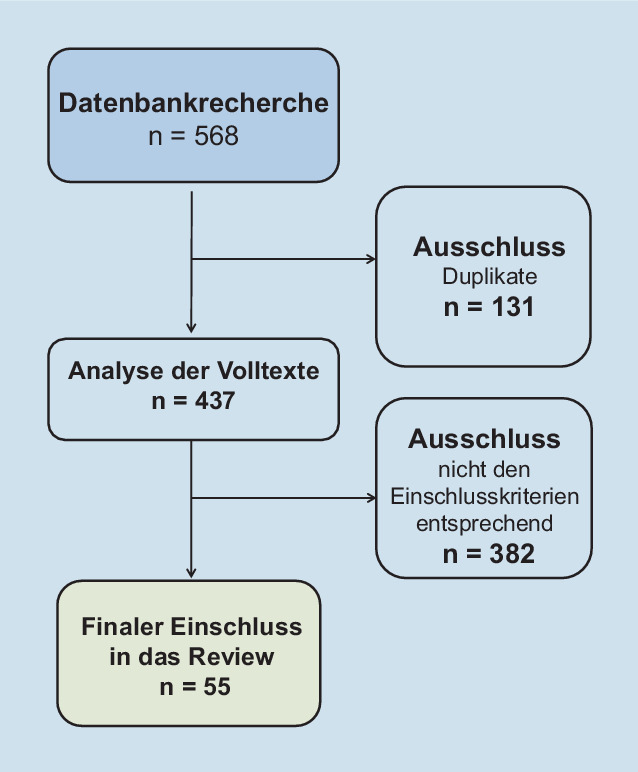

Im vorliegenden Beitrag werden die Einsatzmöglichkeiten sowie Vorteile und Herausforderungen digitaler Tools in der postoperativen Schmerzversorgung vorgestellt und diskutiert. Unter digitalen Tools werden dabei Smartphone- oder webbasierte Anwendungen und der Einsatz von Videobrillen oder virtueller Realität (VR) sowie von künstlicher Intelligenz (KI) verstanden. Die Datenbanken MEDLINE und Web of Science wurden bis zum 15.06.2022 mit den Freitextbegriffen „postoperativer Schmerz“ oder „postchirurgischer Schmerz“ in Kombination mit den Begriffen „digitale Tools“ oder „digital“ oder „virtuelle Realität“ oder „App“ oder „künstliche Intelligenz“ oder „Smartphone“ nach deutsch- oder englischsprachigen Publikationen (Originalarbeiten) durchsucht. Insgesamt ergab die Suche 437 Treffer nach Löschung von Duplikaten (Abb. 1). Zunächst wurden Titel und Zusammenfassung aller gefundenen Zitate auf Einschlusskriterien überprüft und Duplikate entfernt. Alle als relevant erachteten Artikel wurden in die Volltextprüfung aufgenommen. In diesem Schritt wurden erneut die Einschlusskriterien überprüft und Artikel ausgeschlossen, die diese Kriterien nicht erfüllten (beispielsweise Zeitpunkt: präoperative oder intraoperative Phase). Zusätzlich wurden Studien eingeschlossen, die die Nutzbarkeit von digitalen Tools zur postoperativen Schmerzerfassung fokussierten.

Einschlusskriterien:

- Primäres Outcome: Schmerzen/Schmerzintensität wurde(n) bewertet und als Ergebnis berichtet
- Intervention: Nutzung eines digitalen Tools
- Zeitpunkt: postoperative Phase
- Population: Humanstudien, alle Altersstufen
- Artikelarten: randomisierte, kontrollierte Studien (RCT), Pilotstudien, Beobachtungsstudien oder andere Studientypen und Veröffentlichungen mit Originaldaten

Ausschlusskriterien:

- Keine Originaldaten (Übersichten, Metaanalysen, Kommentare, Aktualisierungen, Konsenspapiere)
- Behandlung/Intervention mit digitalen Tools abweichend von den Einschlusskriterien (beispielsweise anderer Zeitpunkt)
- Behandlung/Intervention mit digitalen Tools mit Fokus auf ein anderes postoperatives Symptom (Schmerz nur als sekundäres oder tertiäres Outcome)

Insgesamt wurden 55 passende Publikationen gefunden, die in diese Übersichtsarbeit aufgenommen wurden (vergleiche Online-Zusatzmaterial). Die extrahierten Daten werden zunächst in deskriptiver und thematischer Form nach den Einsatzgebieten (Dokumentation und Erfassung, Selbstmanagement und Information, Schmerzprädiktion, Entscheidungsunterstützung und supportive Schmerztherapie) zusammengefasst. Im deskriptiven Teil werden hiervon 18 beispielhafte Publikationen dargestellt, um die jeweiligen Einsatzgebiete zu verdeutlichen. Den Ergebnissen wird eine Bedeutung beigemessen und diese in Bezug auf Vor- und Nachteile diskutiert.

## Heutige Einsatzgebiete

**Figure 2:**
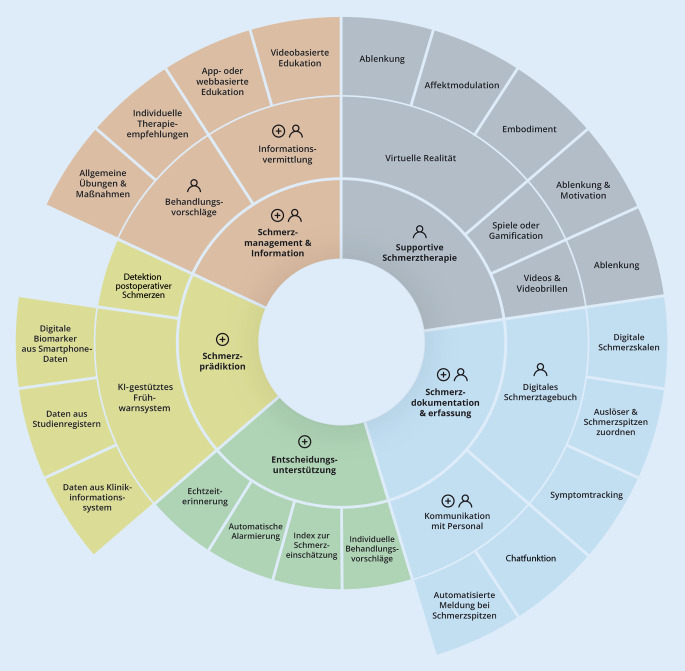

Digitale Anwendungen können im Rahmen der postoperativen Schmerztherapie den Bereichen Schmerzdokumentation und -erfassung, Selbstmanagement, Entscheidungsunterstützung, supportive Schmerztherapie und Schmerzprädiktion zugeordnet werden (Abb. 2). Im Folgenden werden die verschiedenen Einsatzmöglichkeiten aufgelistet und diskutiert. Hierbei zeigen sich Überschneidungen der einzelnen Anwendungsbereiche.

### Schmerzdokumentation und -erfassung

Digitale Tools zur Erfassung postoperativer Schmerzen sind bereits vielfach vorhanden (vergleiche Online-Zusatzmaterial); sie reichen von Smartphone-basierten Versionen von Schmerzskalen [5] bis hin zu Anwendungen, die basierend auf der Schmerzerfassung eine Kommunikation mit dem medizinischen Personal ermöglichen [13]. Während Anwendungen mit einer Dokumentationsfunktion meist nur für Patient:innen konzipiert sind, beziehen andere Anwendungen das Personal als zweite Nutzergruppe mit ein (Abb. 2). Die aktuelle Multicenterstudie AlgoDARPEF zeigte, dass eine App bei einer Nutzungsrate von 70 bis 90 % ein geeignetes Instrument zur Erfassung postoperativer Schmerzen bei Kindern im durchschnittlichen Alter von 6 Jahren ist [31]. Weiterhin hatten einfache Anwendungen zur Dokumentation, beispielsweise elektronische Schmerztagebücher, deutliche Vorteile in Form einer geringeren Erinnerungsverzerrung, einer Datenmessung in Echtzeit und einer geringeren Fehlerquote im Vergleich zu papierbasierten Schmerztagebüchern [30]. Allerdings fehlen auch für diese digitalen Dokumentationsanwendungen derzeit multizentrische RCT auf internationaler Ebene sowie eine breite klinische Umsetzung [30].

Die häufigere Dateneingabe beim Selbstmonitoring verbessert die Einschätzung des Schmerzverlaufs

Je nach Konfiguration einer App besteht der Vorteil für die Patient:innen darin, dass sie selbst entscheiden können, wann sie die Hilfe des medizinischen Personals über die Anwendung in Anspruch nehmen. Dies führte insgesamt nicht zu einer Erhöhung der Belastung des Personals [6]. Die flexible Selbstwahrnehmung und Dokumentation kann so zu einem individuellen, niederschwelligen und bedarfsorientierten Erfassungs- und Kommunikationsverhalten der Patient:innen führen, das nicht an festgelegte Visitenzeiten gebunden ist. Die häufigere und individuelle Dateneingabe beim Selbstmonitoring ermöglicht zudem eine bessere Einschätzung des Schmerzverlaufs und die Zuordnung von Auslösern zu Schmerzspitzen. Werden die Daten beispielsweise an den Akutschmerzdienst weitergeleitet, kann die Schmerztherapie entsprechend individualisiert und zeitig angepasst werden [13]. Solche Anwendungen ermöglichen dem medizinischen Personal ein besseres, schnelleres und realistischeres Verständnis der Patient:innen und ihres Schmerzempfindens. Gleichzeitig führte beispielsweise die Nutzung der App mCare für die Schmerzbeurteilung in der postoperativen Nachsorge und Überwachung der Regionalanästhesie zu einer höheren Zufriedenheit des medizinischen Personals im Vergleich zu einer telefonischen Nachbefragung [13].

Besonders hervorzuheben ist, dass die App-Anwendung Recovery Assessment by Phone Points (RAPP) zur postoperativen Schmerzmessung sogar zu einer Verbesserung der Lebensqualität und zu weniger Angst mit weniger Schmerzen im Wundbereich innerhalb von 14 Tagen im Vergleich zur Standardversorgung führte [14].

### Selbstmanagement und Information

Digitale Tools bieten auch Informationen und Vorschläge zum Umgang mit postoperativen Schmerzen (vergleiche Online-Zusatzmaterial). Die Deutsche Schmerzgesellschaft bietet seit diesem Jahr mit der „Schmerz-App“ für Patient:innen eine App an, die als digitales Nachschlagewerk konzipiert ist und unter anderem Informationen zum perioperativen Schmerzverlauf sowie zu weiterführenden Kontakten, etwa zu Selbsthilfegruppen, bietet. Um einer unzureichenden Akutschmerzbehandlung vorzubeugen, bietet die Österreichische Gesellschaft für Anästhesiologie, Reanimation und Intensivmedizin die App „Perioperatives Schmerzmanagement“ an. Diese unterstützt in erster Linie medizinisches Personal bei der Auswahl und Kombination geeigneter Analgetika und bei der Erstellung multimodaler Behandlungskonzepte.

Der Fokus der App-Entwicklung auf überwiegend edukative Aspekte [16] verschiebt sich mittlerweile hin zu spezifischen Behandlungs- oder Verhaltensvorschlägen für das postoperative Schmerzmanagement von Patient:innen. So konnte die Nutzung der experimentellen PainCoach-App in der akuten postoperativen Phase nach Implantation von Kniegelenkprothesen zu einer Reduktion der Opioideinnahme um 23 % und zur besseren Schmerzkontrolle bei Bewegung führen [22]. Videobasierte edukative Angebote ergänzend zur herkömmlichen Physiotherapie nach Hüftgelenkersatz führten zu einer akuten und bis zu 3 Monate postoperativ anhaltenden Schmerzreduktion um bis zu 2 Punkte auf einer visuellen Analogskala (0–10) in Ruhe und Bewegung [4]. Auch in anderen Bereichen, etwa nach Brustkrebsoperationen, konnte ein einfach zu implementierendes Website-basiertes Angebot mit Videos und Informationen zu Schmerzen, Emotionen, Entspannungsübungen und Verhaltensweisen bereits langfristige postoperative Opioideinsparungen innerhalb von 12 Wochen erzielen [7].

### Entscheidungsunterstützung für Fachpersonal

Entscheidungsunterstützungssysteme in der postoperativen Schmerztherapie sind derzeit meist für das medizinische Personal konzipiert (Abb. 2; vergleiche Online-Zusatzmaterial). Sie dienen dazu, schnell Behandlungsvorschläge zu entwickeln, einen Überblick über komplexe Situationen zu erhalten, potenzielle Risiken zu minimieren und den Nutzen für Patient:innen zu maximieren.

Zur Unterstützung des medizinischen Personals sind intelligente Alarme oder digitale „Echtzeit“-Erinnerungen denkbar. Sie erinnern an wichtige Schritte während der täglichen Arbeit oder helfen, die Flut von Informationen mithilfe von Algorithmen und Risikoanalysen schneller zu bewerten. Hierbei erzielen teils simple Implementierungen im Klinikinformationssystem große Effekte, so beispielsweise eine automatische digitale Erinnerung, die an das intraoperative Bestücken eines gelegten Periduralkatheters (PDK) erinnert. Diese führte zu einem Anstieg angeschlossener PDK im Aufwachraum von 59 auf 85 % [26].

Verschiedene KI-ermittelte Nozizeptionsindizes ermitteln unter anderem die intraoperative physiologische Schmerzreaktion aus Parametern wie der Herzfrequenzvariabilität. Die Indizes können intraoperativ eingesetzt werden und scheinen prädiktiv für APS nach einer Remifentanilgabe bei Ankunft im Aufwachraum zu sein [2]. Zukünftig sind weitere Entscheidungsunterstützungssysteme denkbar, die basierend auf verschiedenen Faktoren dem medizinischen Personal Behandlungsvorschläge unterbreiten.

### Supportive Schmerztherapie: virtuelle Realität, Games und Videos

Digitale Tools werden zunehmend als nichtpharmakologisches Therapieelement eingesetzt und helfen, die Intensität von APS zu lindern sowie Opioide einzusparen [8, 20]. Im Rahmen der supportiven Schmerztherapie werden häufig digitale Therapieelemente wie VR, Videobrillen oder Gamification untersucht [17, 29]. Anwender:innengruppe sind die Patient:innen (Abb. 2). Videobrillen zeigen nur Videos, während VR die Nutzer:innen in eine 3‑dimensionale Welt eintauchen lässt, wobei ein gewisses Maß an Interaktion möglich ist. Bei VR-Anwendungen kommt hinzu, dass sich die Nutzenden in einer technisch-simulierten Umgebung befinden und die dargestellten Inhalte als real wahrgenommen werden (Immersion). Das „Eintauchen“ in die Spielewelt ist in der VR aufgrund ihrer Multidimensionalität besonders relevant für therapeutische Effekte, etwa nach Verbrennungen [18]. Je stärker das Präsenzerleben ist, desto stärker ist die Schmerzreduktion. Bei vielen der Anwendungen basiert der akute analgetische Effekt wahrscheinlich auf Ablenkung [8, 17] und Affektmodulation [18, 29]. Studien über VR vor allem bei chronischen Schmerzen deuten darauf hin, dass das Gefühl, einen eigenen Körper in der VR zu haben (Embodiment), und die Veränderung des Aussehens eines Körperteils die Schmerzerfahrung verändern können [29]. Gleichzeitig können VR-unterstützte Entspannungsübungen nicht nur APS, sondern auch Ängste bei Kindern verringern [20]. Der Einsatz von Spielen oder Gamification, also die Verwendung spielerischer Elemente, kann die Motivation für die Therapie und die Therapietreue erhöhen [18]. Zu den neurobiologischen Mechanismen der Wirkweise von VR ist aktuell wenig bekannt, jedoch scheint eine verstärkte neurophysiologische Veränderung auf der kortikalen Ebene ausgelöst zu werden [18].

Nach derzeitigem Wissensstand können die zuvor beschriebenen digitalen Tools folglich zur Unterstützung der Behandlung akuter und chronischer Schmerzzustände eingesetzt werden. Angesichts des hohen Analgetikaverbrauchs bei Patient:innen mit CPS könnte der Einsatz digitaler Tools in Zukunft Medikamente einsparen [18]. Unklar ist derzeit, wie lange die analgetischen Effekte beispielsweise von VR bei chronischen Schmerzzuständen und insbesondere CPS anhalten. Weitere Forschung im Versorgungsbereich ist erforderlich, um die digitalen Tools in die klinische Praxis und die Regelversorgung flächendeckend integrieren zu können.

### Schmerzprädiktion – Big Data und künstliche Intelligenz

In den letzten Jahren wurden neben den Ergebnissen einzelner RCT zunehmend Daten in großen klinischen Datenbanken erfasst und analysiert. Diese großen Datensätze und deren spezielle Auswertungsansätze, wie die KI-gestützte Analyse von Risikofaktoren, Assoziationen, Clustern und Trends, werden als „Big Data“ bezeichnet. Damit kann eine Vielzahl komplexer Zusammenhänge identifiziert und eine datenbasierte Unterstützung der klinischen Entscheidungsfindung hinsichtlich APS oder CPS ermöglicht werden.

Unter Einbeziehung von 796 Variablen aus einem elektronischen Informationssystem, unter anderem von ICD-10-Diagnosen (*ICD* Internationale statistische Klassifikation der Krankheiten und verwandter Gesundheitsprobleme), demografischen Daten und präoperativen Daten, konnte ein Algorithmus entwickelt werden, der die postoperative Schmerzintensität vorhersagt [28]. Diese Art von automatischer Datenauswertung bietet die Grundlage für individualisierte und vorausschauend eingeleitete Therapien.

Einen Durchbruch für die postoperative Schmerzprävention könnte eine kontinuierliche, digital unterstützte Überwachung im Sinne eines Frühwarnsystems darstellen, das den Übergang von APS zu CPS (zu erwartender Wert auf einer numerischen Rating-Skala [NRS] > 4 nach 3 Monaten) bereits 2 Wochen nach der Operation vorhersagt. Hierbei wurden neben dem Schmerzscore und der präoperativen Behandlung mit Opioiden die Operationsart (beispielsweise Operationen am Knochen) sowie Kälteschmerz und Juckreiz als teils bekannte, teils neue Risikofaktoren im passendsten Modell aufgedeckt und validiert [9].

Ein weiterer innovativer Ansatz im Sinne einer individualisierten Therapie ist die kontinuierliche Erhebung und Analyse digitaler Biomarker der Patient:innen via Smartphone (Abb. 2). Hierfür können beispielsweise Fitnessdaten, das Bewegungsprofil, das Schlafverhalten aus dem Smartphone und ergänzende Angaben zur Schmerzintensität genutzt werden, um postoperative Symptome wie Schmerzen individuell vorherzusagen [19]. Diese Schmerzvorhersage ermöglicht es, individuelle Datensätze ohne weitere technische Hilfsmittel oder das klinische Umfeld in einem nächsten Schritt zu nutzen, um mögliche Behandlungen einzuleiten.

## Vorteile des Einsatzes digitaler Anwendungen in der Schmerztherapie

### Flexibilität und Verfügbarkeit

Digitale Tools bieten zahlreiche Vorteile sowohl für Patient:innen als auch für das behandelnde Personal [3, 8, 9, 15, 18]. Die digitale Datenerfassung zur Schmerzmessung mit Skalen (beispielsweise NRS) kann gleichwertig zur herkömmlichen Methode auf Papier eingesetzt werden [15]. Eine geeignete Variante ist die digitale Schmerzerfassung insbesondere für Kinder, da diese die digitale Selbsterfassung bevorzugen [27].

Digitale Tools ermöglichen eine telemedizinische Betreuung von Patient:innen im häuslichen Bereich

Aufgrund der ständigen Verfügbarkeit können digitale Tools genutzt werden, um Patient:innen unabhängig von Öffnungszeiten einer Arztpraxis zu unterstützen [21]. Zudem ermöglichen sie eine technisch gestützte Betreuung von Patient:innen über eine längere Distanz im häuslichen Bereich (Telemedizin). Dies führt dazu, dass Ressourcen geschont, die Erfahrungen der Patient:innen verbessert und die Patient:innensicherheit erhöht werden [12].

Zudem können Smartphone- oder webbasierte Ansätze individuelle Informationen und Beratung liefern sowie eine kontinuierliche und flexible Dokumentation bieten [13, 14, 16]. Sie ermöglichen eine gezielte Aufklärung, um Informationsdefizite, veraltete Glaubenssätze oder Fehlinformationen, etwa dass man Schmerzen „ertragen müsse“, zu adressieren [21]. Gleichzeitig können digitale Tools das medizinische Personal durch ihren Einsatz bei der Schmerz- und Symptomerfassung sowie bei der Dokumentation entlasten und auf automatisierte Weise individualisierte Behandlungsvorschläge oder Informationen liefern.

### Klinischer Nutzen

Über die reine Schmerzüberwachung und -dokumentation hinaus ist es denkbar, dass digitale Anwendungen einen weiteren klinischen Nutzen erzielen. In einem Pilotprojekt in den USA führte die regelmäßige Nutzung der App „NeuroPath“ nach Wirbelsäulenoperationen zu einer erhöhten Mobilität, einer Verringerung der Schmerzintensität und einer reduzierten Analgetikaeinnahme [12]. Digitale und persönliche Interaktionen mit den Patient:innen könnten synergetisch wirken, positive Behandlungserwartungen könnten verstärkt werden. Auch in Deutschland werden multimodale Therapiekonzepte mit integrierter App erprobt, beispielsweise in der Mulizenterstudie POET-Pain (https://www.poet-pain.de), womit postoperativ und interdisziplinär Patient:innen betreut werden, um Schmerzen zu verhindern.

Ein weiterer möglicher klinischer Nutzen kann die Erkennung von Schmerzen bei Patient:innen sein, die ihre Schmerzen nicht angemessen selbst äußern können, beispielsweise auf der Intensivstation. Hier ist es mittels KI gelungen, zwischen Gesichtsausdrücken zu unterscheiden, die mit unterschiedlichen Schmerzintensitäten verbunden sind [10]. Die KI konnte postoperative Schmerzintensitäten auf einer 11-Punkte-NRS mit einer Sensitivität von über 77 % erkennen, weit besser als die Einschätzung durch geschultes Personal [10]. Eine solche KI birgt zusätzlich Potenzial für Vorhersagemodelle zur personalisierten Behandlung von postoperativem Schmerz. Diese Modelle befinden sich jedoch noch im Entwicklungsstadium .

## Herausforderungen und Grenzen beim Einsatz digitaler Tools in der Schmerztherapie

### Versorgungseffekt

Nicht jedes digitale Tool erzielt automatisch bessere Outcomes als eine konventionelle Methode. So zeigte eine Metaanalyse, dass VR bei Phantomschmerzen keinen Vorteil gegenüber der herkömmlichen Spiegeltherapie brachte [23].

Bei der Einführung neuer digitaler Anwendungen sollte darauf geachtet werden, dass ein positiver Versorgungseffekt erzielt wird oder anderweitige Vorteile, wie ein leichterer Zugang für bestimmte Patient:innenpopulationen (etwa Kinder), entsteht. Aktuelle Entscheidungsunterstützungssysteme liefern oft nur für eine bestimmte Fragestellung valide Informationen. Sie sind oft noch nicht in der Lage, beispielsweise unterschiedliche Therapieziele abzuwägen oder neue unbekannte Entscheidungen zu bearbeiten, wie das Einbeziehen seltener Vorerkrankungen [3].

### Nutzung digitaler Anwendungen – „user-centered design“

Das Nutzungsverhalten bezüglich einer App hängt stark von deren technischer Gestaltung und Benutzerfreundlichkeit ab. App-Anwendungen im postoperativen Setting werden mitunter nur von einem Teil der Patient:innen genutzt. Ein Grund könnte sein, dass die Angebote nicht auf alle Patient:innengruppen zugeschnitten sind („user-centered design“) bzw. ein fehlender Antrieb nach der Operation oder ein stark eingeschränkter Gesundheitszustand (postoperativer Intensivaufenthalt) die Nutzung einschränkt.

Zusätzlich erfordert die Nutzung digitaler Tools eine Schulung der Patient:innen, beispielsweise geriatrischer Patient:innen [24]. Kenntnisse und Vertrauen gegenüber der Technologie gehören ebenfalls zu den wichtigsten Erfolgsfaktoren für eine gelungene Implementierung einer digitalen Gesundheitstechnologie im klinischen Umfeld [24]. Zu wenig Einweisung oder das Auftreten technischer Probleme können das Nutzerverhalten einschränken sowie zu Anwendungsfehlern führen. Anwendungsfehler können auch schädliche Folgen haben, insbesondere für die Gesundheit, so etwa bei falschen postoperativen Belastungen. Bei den Nutzer:innen muss ein Bewusstsein für digitale Gesundheitstechnologien geschaffen werden, und es ist eine Sensibilisierung für digitale Gesundheitskompetenz erforderlich.

Darüber hinaus geht die Einführung einer digitalen Anwendung mit einer Prozessänderung einher, sodass der gesamte Implementationsprozess bedacht werden sollte. In diesem Zusammenhang besteht eine der nichtdigitalen Herausforderungen darin, dass im klinischen Alltag oft wenig Zeit bleibt und digitale Anwendungen nicht als Ersatz für Patient:innenkontakt und Aufmerksamkeit verstanden werden sollten. Digitale Technologien sollten nutzerzentriert konzipiert und implementiert werden, um sie nachhaltig und nutzbringend in der Versorgung zu verankern.

### Technische Ausstattung

Beim Einsatz digitaler Tools ist die allgemeine technische Ausstattung in der Klinik und bei den betroffenen Patient:innen zu berücksichtigen, unter anderem der Internetempfang in der Klinik und im häuslichen Umfeld. Darüber hinaus müssen Datenschutz und aktuelle Sicherheitsstandards berücksichtigt und überprüft werden [30], Datenschutzkonzepte und Sicherheitsmechanismen sollten frühzeitig berücksichtigt und integriert werden, beispielsweise der Schutz vor Hackerangriffen. Insbesondere bei der Einführung und Erweiterung bestehender Programme sollte auf die Interoperabilität der Systeme, etwa zwischen Klinikinformationssystem und App, geachtet werden. Neben der technischen Ausstattung sollten auch die Kosten für Entwicklung, Weiterentwicklung sowie Unterhaltung einer digitalen Anwendung bedacht werden.

## Ausblick

Digitale Tools erfüllen eine wesentliche Funktion auf dem Weg zu einer individualisierten und personalisierten Medizin im postoperativen Schmerzmanagement. Sie bieten das Potenzial, einen Beitrag zur Optimierung der postoperativen Schmerztherapie zu leisten, insbesondere im weniger betreuten häuslichen Umfeld. In Zukunft könnte sich das postoperative Schmerzmanagement von starren Behandlungs- und Analgetikaschemata wegbewegen. Vielmehr ist ein individuelles Schmerzfrühwarnsystem denkbar, das eine Vielzahl von Parametern wie das Verhalten der Patient:innen und die Art der Operation kontinuierlich bewertet und berücksichtigt. Auf diese Weise kann Wissen vermittelt und die Kommunikation mit den Behandler:innen schnell und gezielt hergestellt werden.

Darüber hinaus lassen digitale Tools neue Ansätze für die Vernetzung interdisziplinärer Teams erwarten, um über große Entfernungen zeitlich flexibel miteinander zu kommunizieren. Würden dem medizinischen Personal mehr digitale Tools als Teil der Therapie zur Verfügung stehen, könnten nach vielversprechenden ersten Studienergebnissen postoperative Schmerzen zu einem relevanten Anteil gelindert und Opioide eingespart werden. Dies würde die klinische Arbeit erleichtern und weitere Therapieoptionen bieten. Zu bedenken ist, dass Schulung und Weiterbildung von Personal und Patient:innen eine Voraussetzung für die erfolgreiche Implementierung digitaler Tools in einen Behandlungsprozess sind.

Die digitalen Lösungen sollten aus der Entwicklungsphase in den klinischen Alltag integriert werden

Künftige Studien und Projekte sollten dazu beitragen, die vielversprechenden Forschungsansätze in verschiedenen Patient:innenpopulationen und Realitäten zu überprüfen. Zusätzlich sollten die digitalen Lösungen aus der Entwicklungs- und Forschungsphase in den klinischen Alltag integriert werden, wo Endnutzer:innen in das Design und die Implementierung einbezogen werden können. Interoperabilitätsstandards sollten berücksichtigt werden, und die Integration der Tools in bestehende Lösungen, wie etwa Patient:innenportale, sollte geprüft und angestrebt werden. Die Nutzung digitaler Tools für das Management von CPS ist noch weitgehend unerforscht. Es bleibt abzuwarten, ob sich die bisherigen Erkenntnisse übertragen lassen oder sogar neue Potenziale bieten. Zudem sollte die Entwicklung künftiger digitaler Tools nicht nur auf einzelne Komponenten des postoperativen Schmerzes ausgerichtet sein, sondern ganzheitliche Ansätze für den Behandlungsprozess auf Grundlage der komplexen Ätiologie verfolgen.

## Fazit für die Praxis

- Digitale Tools kommen bereits heute zum Einsatz und haben großes Potenzial, die postoperative Schmerztherapie sowohl für Patient:innen als auch für klinische Anwender:innen zu optimieren.
- Mit Smartphone- und webbasierten Applikationen können Patient:innen ihre postoperativen Schmerzen selbst erfassen und managen, während das Fachpersonal ein besseres Verständnis für den Schmerzverlauf und individualisierte Entscheidungshilfen erhält. Erste Pilotprojekte zur Frühwarnung und -erkennung akuter postoperativer Schmerzen oder einer möglichen Chronifizierung liefern vielversprechende Ergebnisse.
- Der Einsatz digitaler Tools wie der virtuellen Realität zeigt relevante klinische Ergebnisse, wie die Reduktion postoperativer Schmerzen und des Opioidverbrauchs.
- Es bedarf weiterer Versorgungsforschung, um digitale Tools, die heute noch oft Modellstatus haben, in die klinische Praxis zu integrieren. Hierbei sind eine nutzerzentrierte Implementierung, digitale Kompetenzen der Anwender:innen und die Verzahnung von Entwickler:innen und Endnutzer:innen essenziell.

## Supplementary Information




