# Supplementary material for: Application possibilities of digital tools in postoperative pain therapy
Source: Schmerz. 2023 Jul 10;37(4):234–41. [Article in German] doi: 10.1007/s00482-023-00732-7 (PMC10368541; doi:10.1007/s00482-023-00732-7)
Supplement: Supplementary file 1 [file 482_2023_732_MOESM1_ESM.pdf]

Online-Zusatzmaterial

Tab. 1 Übersicht der eingeschlossen Studien
* = im Text erwähnt

Autor:innen	Jahr	Bevorzugte Anwendergruppe	Digitales Tool	Journal
**Dokumentation & Erfassung**				
Carlier et al.	2021	Erwachsene	App - Smartphone	Anaesthesia, Critical Care & Pain Medicine
Carrier et al.	2016	Erwachsene	Textnachrichten	Journal of Visceral Surgery
Cheng et al.	2021	Erwachsene	Textnachrichten	Journal of Thoracic Disease
Chiu et al. *	2019	Erwachsene	App - Smartphone	Canadian Journal of Anaesthesia
Dahlberg et al.*	2019	Erwachsene	App - Smartphone	International Journal of Surgery
Drake-Brockman et al.	2022	Kinder	Textnachrichten	Paediatric Anaesthesia
Highland et al.*	2019	Erwachsene	App - Smartphone	Pain Medicine
van Hout et al.	2020	Erwachsene	App – Tablet & Smartphone	Hernia: The Journal of Hernias and Abdominal Wall Surgery
Jaensson et al.*	2017	Erwachsene	App - Smartphone	British Journal of Anaesthesia
Kane et al.	2020	Erwachsene	Webbasierte Plattform	Journal of Shoulder and Elbow Surger
Khanwalkar et al.	2019	Erwachsene	Webbasierte Plattform	International Forum of Allergy & Rhinology
Ooi et al.	2021	Erwachsene	Textnachrichten	Journal of Medical Systems
Schiavo et al.	2022	Erwachsene	App - Smartphone	PloS One
Slepian et al.	2020	Erwachsene	Webbasierte Plattform	Digital Health
Sood et al.	2020	Erwachsene	App - Smartphone	Plastic and Reconstructive Surgery Global Open
Sun et al.	2015	Kinder	App - Smartphone	Paediatric Anaesthesia
Thiel et al.	2020	Erwachsene & med. Personal	App - Smartphone	PLOS ONE
Thiel et al.	2022	Medizinisches Personal	App - Smartphone	JMIR Human Factor
Tiozzo et al.	2021	Kinder & Eltern	App - Smartphone	Journal of Child Health Care
Walrave et al.*	2022	Eltern & Kinder	App - Smartphone	PAIN
**Schmerzmanagement & Information**				
Birnie et al.	2018	Kinder	App - Smartphone	Canadian Journal of Pain
Darnall et al.*	2019	Erwachsene	Webbasierte Plattform	Pain Medicine
Dotto et al.	2020	Erwachsene	App - Smartphone	Canadian Journal of Anaesthesia
Glauser et al.*	2019	Erwachsene	App - Smartphone	Mhealth
Newton et al.	2017	Kinder	App - Smartphone	International Journal of Pediatric Otorhinolaryngology
Pizzi et al.	2020	Erwachsene	Digitaler Medikamentenspender	Orthopedic Nursing
Sun et al.*	2018	Eltern & Med. Personal	App - Smartphone	Paediatric Anaesthesia
Timmers et al.	2019	Erwachsene	App - Smartphone	JMIR MHealth and UHealth
Weyns et al.	2020	Erwachsene	Digitale Schmerzpumpe	Acta Anaesthesiologica Belgica

**Entscheidungsunterstützung**				
Momtahan et al.	2007	Medizinisches Personal	Algorithmus-basierte Software	Studies in Health Technology and Informatics
Pronk et al.*	2020	Erwachsene	App - Smartphone	JMIR MHealth and UHealth
Shah et al.*	2019	Medizinisches Personal	Anästhesie-Informations-Management-System	Anesthesia & Analgesia
**Supportive Schmerztherapie**				
Binay et al.	2022	Kinder	Virtuelle Realität	Journal of Perianesthesia Nursing
Cardenas et al.	2021	Kinder	Virtuelles Rehabilitationsspiel	Developmental Neurorehabilitation
Cetinkaya et al.*	2022	Erwachsene	Videos	Scientific Reports
Ding et al.*	2019	Erwachsene	Virtuelle Realität	The Journal of International Medical Research
Dings et al.	2021	Erwachsene	Virtuelle Realität	BJU International
Gao et al.	2021	Kinder	Virtuelle Realität	National Journal of Andrology
Gray et al.	2021	Erwachsene	Virtuelle Realität	Otolaryngology--Head and Neck Surgery
Hansen et al.	2015	Erwachsene	App - Tablet	BMC Complementary and Alternative Medicine
Hou et al.	2019	Erwachsene	App - Smartphone	JMIR MHealth and UHealth
House et al.	2016	Erwachsene	Virtuelles Rehabilitationsspiel	British Journal of Pain
Mosso Vázquez et al.	2019	Erwachsene	Virtuelle Realität	Annual Review of CyberTherapy and Telemedicine
Olbrecht et al.*	2021	Kinder	Virtuelle Realität	Journal of Medical Internet Research
Olbrecht et al.	2021	Kinder	Virtuelle Realität	Pain Medicine
Pandrangi et al.	2022	Erwachsene	Virtuelle Realität	JAMA Otolaryngology–Head & Neck Surgery
Pandya et al.	2017	Erwachsene	Virtuelle Realität	Korean Journal of Anesthesiology
Rothgangel et al.	2018	Erwachsene	Erweitere Realität	Clinical Rehabilitation
Rothgangel et al.	2019	Erwachsene	Erweitere Realität	Clinical Rehabilitation
Specht et al.	2021	Kinder	Virtuelle Realität	The American Surgeon
**Schmerzprädiktion**				
Boselli et al.*	2014	Medizinisches Personal	Berechneter Index	British Journal of Anaesthesia
van Driel et al.*	2022	Medizinisches Personal	Künstliche Intelligenz	British Journal of Anaesthesia
Fontaine et al.*	2022	Medizinisches Personal	Künstliche Intelligenz	European Journal of Pain
Low et al.*	2021	Medizinisches Personal	Künstliche Intelligenz	JMIR Cancer
Tighe et al.*	2015	Medizinisches Personal	Künstliche Intelligenz	Pain Medicine
